# In-vivo dosimetry with Gafchromic films for multi-isocentric VMAT irradiation of total marrow lymph-nodes: a feasibility study

**DOI:** 10.1186/s13014-015-0391-y

**Published:** 2015-04-12

**Authors:** Pietro Mancosu, Pierina Navarria, Giacomo Reggiori, Luca Cozzi, Antonella Fogliata, Anna Gaudino, Francesca Lobefalo, Lucia Paganini, Valentina Palumbo, Barbara Sarina, Antonella Stravato, Luca Castagna, Stefano Tomatis, Marta Scorsetti

**Affiliations:** Radiation Oncology Department, Humanitas Clinical and Research Center, Rozzano Milan, Italy; Bone Marrow Transplantation Unit, Humanitas Clinical and Research Center, Rozzano Milan, Italy

**Keywords:** Total marrow irradiation, In vivo dosimetry, Bone transplantation, GafChromics, Plan optimization, Radiotherapy

## Abstract

**Background:**

Total marrow (lymph-nodes) irradiation (TMI-TMLI) by volumetric modulated arc therapy (VMAT) was shown to be feasible by dosimetric feasibility studies. It was demonstrated that several partially overlapping arcs with different isocenters are required to achieve the desired coverage of the hematopoietic or lymphoid tissues targets and to spare the neighbouring healthy tissues. The effect of isocenter shifts was investigated with the treatment planning system but an in- vivo verification of the procedure was not carried out. The objective of this study was the in-vivo verification of the consistency between the delivered and planned doses using bi-dimensional GafChromic EBT3 films.

**Methods:**

In a first phase a phantom study was carried out to quantify the uncertainties under controlled conditions. In a second phase three patients treated with TMLI were enrolled for in-vivo dosimetry. The dose prescription was 2Gy in single fraction. Ten arcs paired on 4-6 isocenters were used to cover the target. Cone Beam Computed Tomography (CBCT) was used to verify the patient positioning at each isocenter. GafChromic EBT3 films were placed below the patient on the top of a dedicated immobilization system specifically designed. The dose maps measured with the EBT3 films were compared with the corresponding calculations along the patient support couch. Gamma Agreement Index (GAI) with dose difference of 5% and distance to agreement of 5 mm was computed.

**Results:**

In the phantom study, optimal target coverage and healthy tissue sparing was observed. GAI(5%,5 mm) was 99.4%. For the patient-specific measurements, GAI(5%,5 mm) was greater than 95% and GAI (5%,3 mm) > 90% for all patients.

**Conclusions:**

In vivo measurements demonstrated the delivered dose to be in good agreement with the planned one for the TMI-TMLI protocol where partially overlapping arcs with different isocenters are required.

## Background

Total body irradiation (TBI) is adopted as a part of conditioning regimen for patients who undergo hematopoietic cell transplantation in multiple myeloma, leukaemia and lymphomas [1,2]. The patients are usually positioned at around 3-4 meters from the LINAC gantry using open field aperture in order to obtain homogeneous dose distribution to the target. A direct consequence is the difficulty of sparing normal tissue while maintaining the full dose to the target. Physical blocks can be used to shield lungs, kidneys, and other critical organs but, in this way, a local dose reduction to the hematopoietic target may occur. In this context, the inverse optimization procedure of modern treatment planning systems offers the opportunity to spare the organs at risk (OARs) and neighbour healthy tissues, while maintaining the best target coverage. This technique was tested for these patients, changing from the TBI concept to a selective total marrow (and lymph-nodes) irradiation (TMI-TMLI). The most important clinical reason to use TMI-TMLI instead of conventional TBI is related to the possibility of delivering radiation on bone marrow and lymphopoietic tissue more accurately without exceeding in toxicity on radiosensitive organs such as liver, lung, bowel, and kidney, as reported by Kim et al. over more than 100 patients [3].

Dosimetric study on conventional LINAC using intensity modulated radiotherapy (IMRT) [4,5], helical Tomotherapy (HT) based approaches [6,7], and more recently, Volumetric Modulated Arc Therapy (VMAT) using RapidArc (RA) approach (Varian Medical Systems, Palo Alto, CA) [8-13] showed the feasibility of TMI-TMLI from a planning point of view. Based on these studies, starting in October 2010, TMI-TMLI has been delivered in our institute by means of RA. In particular, TMI (6 fractions of 2 Gy, 2 times per day) was adopted for patients who underwent autologus transplantation, while a single dose of 2 Gy TMLI was adopted for patients who underwent to haploidentical transplantation with nonmyeloablative conditioning regimen. It is well known that there is a direct correlation between delivered dose and outcome. In a randomized study conducted some years ago, Clift et al. demonstrated that higher dose TBI (15.75 Gy) was associated to a reduced relapse incidence with respect to the standard 12 Gy [2]. However, this was not associated with a better survival because of enhanced treatment related mortality. The possibility to selectively deliver radiotherapy to bone marrow and/or lymphopoietic tissue, sparing other sensitive organs, could raise the therapeutic index of radiotherapy. Recently, Bornhauser and colleagues compared TBI 8 Gy to TBI 12 Gy before allergenic transplantation, and no difference was found in terms of outcome [14]. Few years ago, the Seattle team proved that TBI dose can be further reduced down to 2 Gy. This dose allowed, with an appropriate immune-suppression, the achievement of a mixed chimerism observed in mouse model [15] and confirmed in human studies [16]. For a deeper argumentation regarding the two schemes we refer to bibliography.

In our institute, all modulated plans (i.e. IMRT and VMAT) are verified before the first fraction by a specific pre-treatment quality assurance (QA). In particular, for the first 5 TMI-TMLI patients we measured the integral dose of each single arc with three different methodologies: MatriXX (IBA Dosimetry) [17], EPIQA (Epidos) [18], and GafChromics. The plan accuracy was evaluated by GAI(3%,3 mm). Since no substantial differences were found between the three measurements, we decided to start verifying the TMI-TMLI plans using only EPIQA. However, in this way it is possible to check the accuracy of each single arc, though the contributions from different arcs cannot be directly evaluated. In fact, several partially overlapping arcs with different isocenters are required to achieve the desired coverage of the TMI-TMLI target and to spare the neighbouring healthy tissues.

In vivo dosimetry is usually applied in external beam RT to measure differences between planned and delivered dose [19]. This is generally performed by placing some type of detector on the skin or close to that part of the patient anatomy in which the dose has to be measured. Detectors for in vivo dosimetry can be divided into real-time and passive ones. For a deeper discussion about the characteristics and advantages, we refer to the review by Mijnheer [19]. Historically, in vivo dosimetry for TBI treatments was performed to ensure proper delivery of the intended radiation dose throughout the body. At this purpose, many detectors were tested in TBI, including Termoluminescent detectors (TLD), Metal Oxide-silicon Semiconductor Field Effect Transistor (MOSFET), and GafChromics, reporting dose uniformity within 10% over the body [20-22]. In our study we decided to use GafChromics EBT3 films because they allow the detection of large field areas with an adequate dose response in terms of energy dependence, linearity, and reproducibility. GafChromics EBT3 are radiation-induced auto-developing photon and electron-beam analysis films and are available for therapeutic radiation dosimetry in radiotherapy applications to provide accurate in-vivo dosimetry measurements [23,24]. In particular, the ability to offer a bidimensional evaluation of the dose, is an attractive option for TMI-TMLI in-vivo dosimetry as the integral delivered dose is originated by different arcs with separate isocenter positions. Film flexibility allows to easily position it under the patient’s body at the surface of the treatment couch.

In our previous work we simulated small motions on TPS and we evaluated the dosimetric consequences without in vivo measurements [13]. The aim of the current study is to perform in vivo measurements for patients that underwent TMI-TMLI using GafChromic EBT3. Particular attention was paid in verifying the correct dosimetric junction from fields with different isocenters as the case of total marrow irradiation by VMAT. Data from phantom and patients were included into the analysis.

## Methods

The study was divided into two parts. In the first part, the use of GafChromics for pre-treatment dosimetry of plans with multi-isocenter geometry was tested in a phantom. The second part included in vivo measurements on three patients undergoing TMI-TMLI treatment.

### Feasibility study on phantom

A homogeneous water equivalent phantom with exterior dimensions of 31.4 cmD × D34 cmD × D22 cm (MULTIcube - IBA Dosimetry) and with a removable film cassette for independent verification was scanned with a 16 slice computed tomography (CT) system (Brillance CT Big Bore - Philips Medical System). A complex target with volume >2000 cm^3^ with a central hole was manually generated. A plan composed of two 6 MV arcs with different isocenters was optimized. Collimator rotation was set to 90°. Field width was set to 40 cm while field length ranged from 11 to 19 cm. Dose prescription was 2 Gy in single fraction to the target. Plan objectives were: V98% > 98% to the target (i.e. 98% of the target volume should receive at least 98% of the prescription dose) and to minimize the volume receiving more than 50% of the prescription dose (i.e. 1 Gy).

The plan was generated using the progressive resolution optimizator algorithm PROIII version 10 (Varian Medical Systems, Palo Alto, CA). This version allows the simultaneous optimization of a maximum of 10 full arcs. All dose distributions were computed with the Analytical Anisotropic Algorithm (AAA, version 10.0.28) implemented in the Eclipse planning system with a calculation grid resolution of 2.5 mm. The plan was designed and optimized for a Varian TrueBeam equipped with a Millennium MLC with leaf width of 5 mm at the isocenter in the central part, up to 20 cm and 10 mm for the external part.

Cone beam CT (CBCT) image guidance with on-line couch adjustment was carried out for the two isocenters before the delivery, using an action level of 1 mm. Couch repositioning was operated after automatic matching of CBCT images to reference planning CT based on the phantom edges, followed by manual refining. The film was placed inside the cassette along the coronal plane.

### Pilot study on patients

Between October 2010 and November 2014, 25 patients candidate to hematopoietic cell transplantation in multiple myeloma, leukaemia and lymphomas with mielo-riductive or ablative intent received TMI or TMLI treatment by VMAT approach at Humanitas Cancer Center. In particular, mielo-reductive bone marrow transplantation uses low doses (usually 2 Gy) which do not destroy the host bone marrow but suppress the host immune system sufficiently to promote donor engraftment. In this pilot study, the last three patients (all TMLI with mielo-reductive intent) were enrolled.

**Figure 1:**
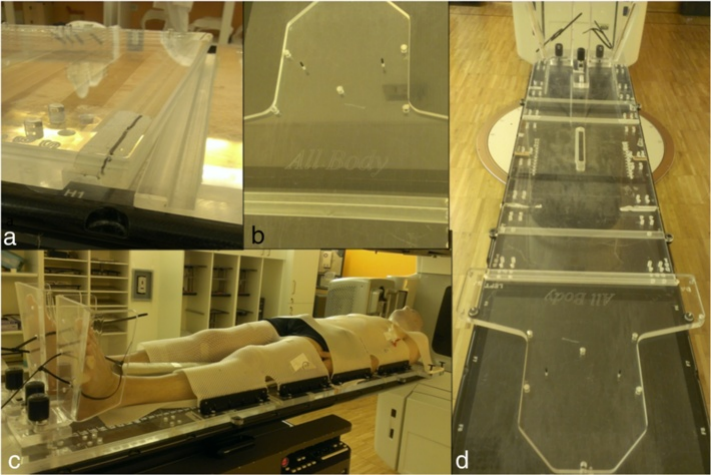
All Body frame immobilization support. (**a**) junctions between the successive boards; (**b**) head and neck frame; (**c**) Patient position during the TMI delivery using the All Body frame; (**d**) The three frames mounted on the couch before patient positioning.

Planning CT scans extended from the top of the skull to the knees. CT was acquired with 3-mm slice in a free breathing mode (head first supine). Arms were placed on the couch, along the body, as close as possible to the body, and with the fingers under the glutei to help patient positioning reproducibility. Patients were positioned using a dedicated immobilization system developed by the radiotherapy technicians (RTT) team to best fix the patient (internally named “All Body frame” – see Figure 1) [13]. Briefly, it consists of three successive frames that can be coupled and linked up to a length of 200 cm to obtain the “All Body frame”. The patient is then positioned over the frames and personalized masks are used to best fix the patient. This prototype has a thickness of 2 cm of Plexiglas® that has to be accounted for during dose calculation due to its non-negligible X-ray attenuation.

The planning target volume (PTV) was defined as all the bones with exclusion of the mandible and maxillary structures, providing a generous margin around the bone marrow. The whole chest wall was considered as part of the PTV to include the breathing motion of the ribs. Furthermore, the bones of arms and legs were enlarged by 5-10 mm to account for possible involuntary motion. The spleen and lymph-nodes, plus isotropic margin of 5 mm on the three directions, were included into the PTV.

OARs were delineated by the radiation oncologist, using, where possible, automatic and semiautomatic tools. The structures considered in the study were: eyes, lenses, parotids, oral cavity, thyroid, trachea (including the esophagus), lungs, heart, stomach, kidneys, liver, bowel cavity, bladder, rectum and genitals.

Dose prescription to the PTV was a single delivery of 2 Gy plus an eventual boost of 2-6 Gy based on target delineated on positron emission tomography (PET). Dose was normalized to PTV- V_98%_ = 98% in order to cover the target with full dose.

PTV planning objectives aimed to limit minimum and maximum doses. No specific dose-volume planning objectives were defined for the OARs. Plans were designed in order to maximize the sparing of each OAR and to reduce median doses (D_50%_) below the 50% of the prescription dose [8].

The plans were optimized according to anatomy driven strategy proposed in a previous work [9]. Briefly, 10 full arcs (360°) of 6 MV were optimized simultaneously with 4-6 isocenters using asymmetric jaw settings to cover the entire PTV length. Both isocenters positions and jaw sizes were chosen according to the individual anatomy. These parameters were optimized in order to minimize the target volume near the field edges (i.e. to maximize the freedom of motion of MLC leaves inside the field aperture, for example avoiding arcs with ribs and iliac wings in the same BEV). In particular cases, two isocenters placed on the arms were added to better cover the target. For example this was required in case of fat patients, or patients with articular dysfunctions. Verification by CBCT was performed for each isocenter before the delivery. Therefore up to 6/8.

CBCT were acquired for each session. For the Varian onboard imaging system OBI, CT dose index (CTDI) was estimated to be 2.5 cGy and 0.7 cGy for, respectively, pelvis and thorax acquisitions [25]. The new XI imaging available on Truebeam platform further reduced the CTDI. Therefore we considered as adequate this very particular procedure. However, it cannot be considered as a standard practice. Bony anatomy-based alignments were performed: for the thoracic region, particular attention was paid to the chest wall over the arms considered that a bigger margin is used for the arms. An action level of 1 mm was adopted.

### Dosimetric analysis of GafChromic films

For the phantom study, the GafChromic EBT3 film was placed at the isocenter level in the removable film holder along the coronal plan.

**Figure 2:**
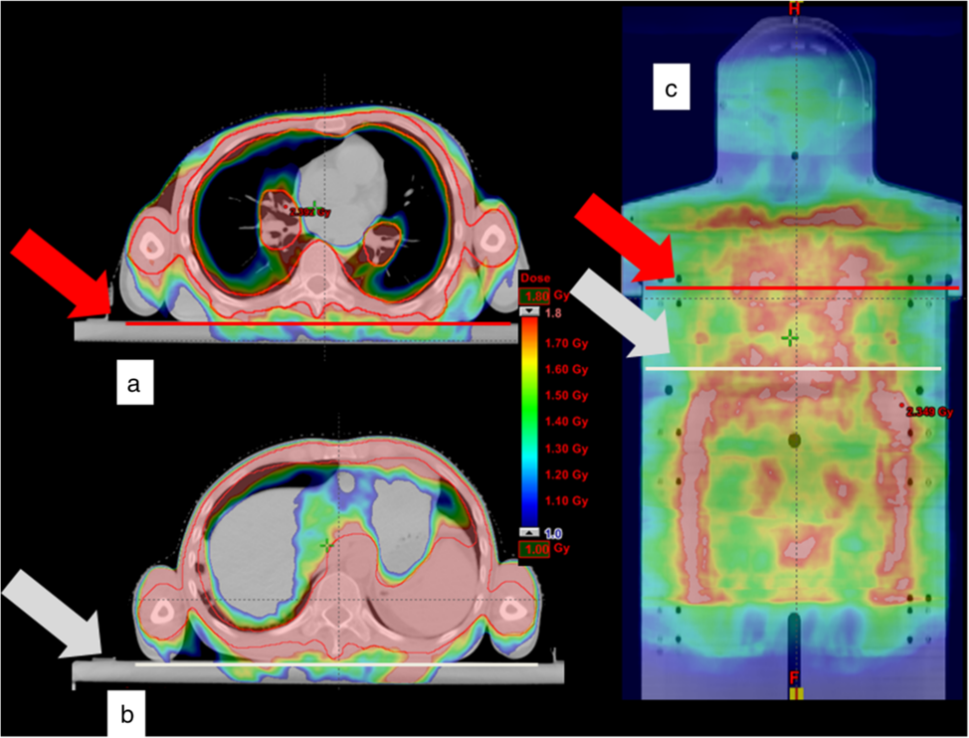
Example of dose distribution calculated by Eclipse for a TMLI patient. Two axial (**a** and **b**) and one coronal (**c**) views are reported. The black squares in the coronal view show the position of the GafChromics for in vivo dosimetry. The white and red lines and arrows in **a**) and **b**) represent the y coordinates where films were positioned. The lines in **c**) represent the z coordinates of the axial views shown in (**a**) and (**b**). Color-wash ranging from 50% to 90% was used as look-up-table.

For each patient, GafChromic EBT3 films were placed above the patient on the top of the dedicated immobilization system (“All Body Frame”) in correspondence of the overlapping regions (see Figure 2). This support is 2 cm Plexiglas thick. Therefore, as the thickness is more than the build-up depth (i.e. 1.5 cm for 6MV beam), the evaluation on the GafChromic is a dose measurement.

Particular attention was paid on fixing the films in recognizable coordinates to facilitate the successive GAI evaluation.

The EBT3 films were processed with an Epson10000XL scanner and compared with the TPS- calculated dose along the patient support (see Figure 2), using I’mRT OmniPro software (Scanditronix). For further information about absolute dose calibration we refer to the bibliography [23,24].

Gamma Agreement Index (GAI), scoring the percentage of modulated area computed with dose difference (d) of 5% and Distance To Agreement (DTA) of 5 mm, was determined. Furthermore, d and DTA of, respectively, 3% and 3 mm were calculated (i.e. GAI (3%, 3 mm), GAI (3%, 5 mm), GAI (5%, 3 mm)) to quantify the relative contribution of the two variables to the gamma value [26].

## Results

### Feasibility study on phantom

**Figure 3:**
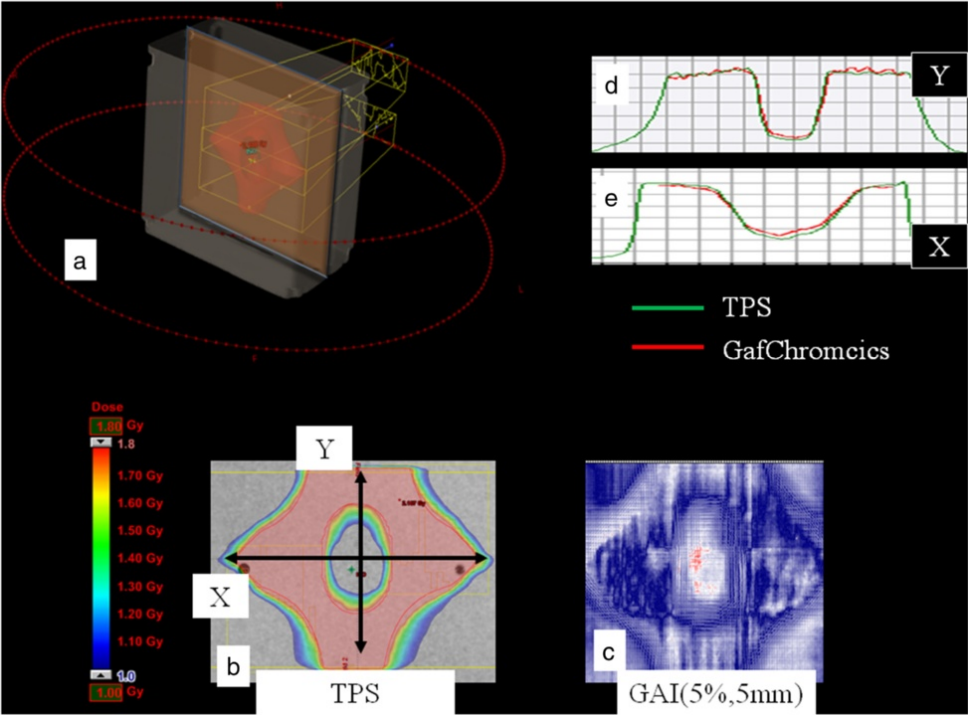
Dose distribution for the plan optimized on the phantom. In (**a**) the measurement setting of the solid phantom and the inserted GAF is shown. Dose distribution (range 50%-90%) calculated by the TPS for the phantom plan with the overlap is reported in (**b**). (**c**): GAI (5%, 5 mm) map related to phantom plan: in red are reported the points that didn’t pass the test (i.e. Δdose and DTA exceeded the threshold values of 5% and 5mm respectively). In (**d**-**e**) the X and Y profiles for the TPS (green) and the Gafchromic (red) are reported.

Figure 3 shows the dose distribution for the plan optimized on the phantom. The figure also reports the GAI (5%, 5 mm) and the profiles along X and Y axis for the treatment planning system (TPS) and the delivered dose estimated with GafChromic dosimetry. In terms of planned dose distribution, target coverage was optimal. Regarding the GafChromic dosimetry evaluation, GAI (5%, 5 mm) = 99.7% was found. The first two rows of Table 1 report the data of the GAI using d of 3/5% and DTA of 3/5 mm. In particular, GAI (5%, 3 mm) was greater than 95%, while GAI (3%, 5 mm) was much lower, revealing that the results are more affected by the dosimetric uncertainties than the geometrical ones.

**Table 1:** **Data on the GAI using Δd of 3/5 mm and DTA of 3/5 mm for phantom study, and patients with the region in which the GafChromics are placed**

**GAI**	**5 mm 5% [%]**	**3 mm 5% [%]**	**5 mm 3% [%]**	**3 mm 3% [%]**
PhantomGAP yes	99.4	95.6	87.1	75.2
Pz 1 H&N	96.2	92.5	82.5	74.0
Pz 1 Abdomen	96.3	90.0	79.2	67.6
Pz 2 H&N	95.3	88.7	77.3	64.4
Pz 2 Abdomen	98.4	97.6	84.9	79.5
Pz 3 H&N	96.3	89.7	79.3	68.4

### Pilot study on patients

For the patients study, the three TMLI plans achieved all the dosimetric goals on PTV and Healthy Tissues described in the internal protocol and previous studies [8,9,13]. Regarding the in-vivo dosimetry, GAI (5%, 5 mm) was greater than 95% for the five evaluations (see Table 1).

**Figure 4:**
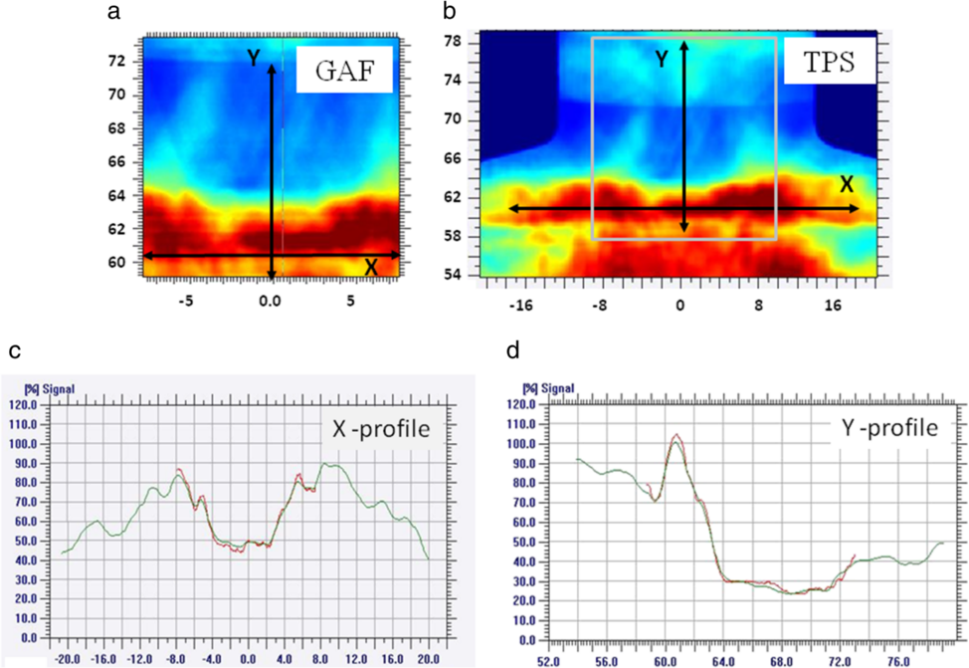
In vivo measurement on the neck region (15 cm × 15 cm) for the first patient. Planar dose distribution for (**a**) the GafChromic and (**b**) the TPS for patient 1. In (**c**-**d**): profiles along the X and Y axis for the TPS (green) and the Gafchromic (red) are reported.

Figure 4 illustrates the analysis of the neck region (15 cm × 15 cm) for the first patient. In particular, the dose maps measured by the GafChromic EBT3 and planned by TPS (Eclipse – Varian) are shown. The comparison of the two dose profiles is reported along the two cardinal directions (X, Y), presenting a GAI (5%, 5 mm) of 96.2%. The figure shows also the dose from each arc along Y direction calculated by the TPS, proving the complicate composition of the integral dose.

**Figure 5:**
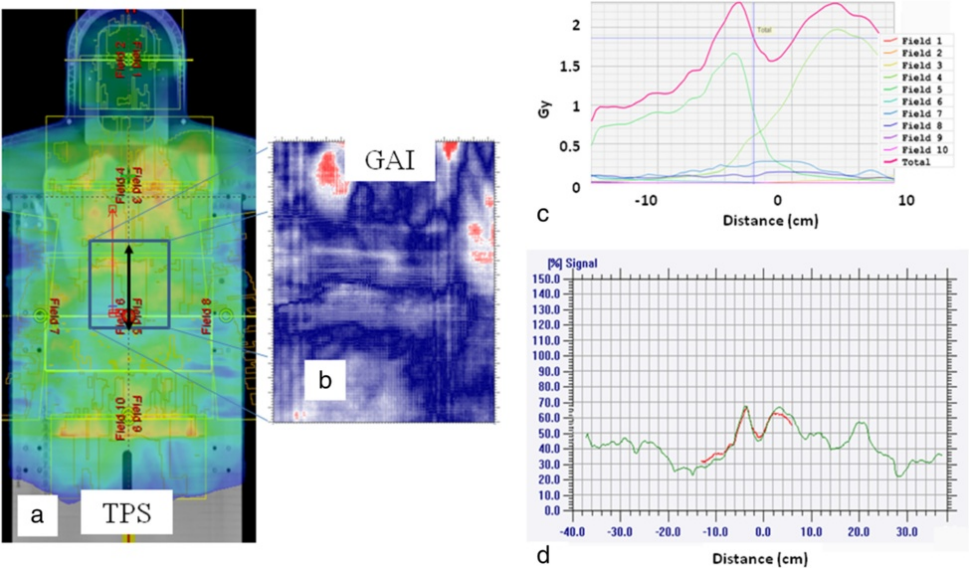
In vivo measurement on the abdomen region (20 cm × 15 cm) for the second patient. (**a**) Planar dose distribution calculated by TPS for patient 2; in particular, the region where the GafChromic was acquired is outlined with a rectangle. (**b**) GAI (5%, 5 mm) evaluation with same scale as Figure 3. (**c**): integral dose profile along Y axis calculated by TPS with single arc contributions (4 different arcs for the present case). In (**d**) Profiles along the Y axis for the TPS (green) and the Gafchromic (red) are reported.

Figure 5 illustrates the analysis of the abdominal region (20 cm × 15 cm) for the second patient, in which the dose derived from four different arcs (two isocenters centered along the medial positions and the and the other two centered on the two arms). In this case. the GAI (5%, 5 mm) was 98.4%.

## Discussion and conclusions

TMI-TMLI is a relatively new approach for treating patients who undergo hematopoietic cell transplantation in multiple myeloma, leukaemia and lymphomas and for reducing the toxicities induced after the conventional TBI treatments. In particular, the cranial-caudal (C-C) extension of the patient is much greater than the jaws of the linac and requires the use of multi-isocenter approach to treat TMI-TMLI. At this purpose, to simplify the repositioning between successive isocenters, in our centre, the isocenters are usually placed using the same Anterior-Posterior and Left-Right coordinates, and changing only the C-C direction. Therefore the total dose is the sum of many doses delivered with different isocenter positions, creating a non-trivial situation and in-vivo dosimetry could help in understanding the reliability level of this procedure. Surucu and colleagues, using an anthropomorphic phantom and thermoluminescent detectors (TLD), demonstrated that VMAT is safe, accurate and efficient in delivering TMI-TMLI in the junction regions, where the dose comes from two different arcs [12]. Our study on phantom confirmed their results using an independent method. These results confirmed what was theoretically demonstrated in our previous study in which the dosimetric consequences of inaccurate isocenter positioning during treatment of TMI-TMLI using VMAT were evaluated [13]. In that paper, two patients were considered and three series of random shifts were applied to the 5 isocenters in order to simulate involuntary patient motion during treatment. The shifts were applied separately in the three directions. We demonstrated that the correct isocenter repositioning of TMI-TMLI patients is fundamental, in particular in C-C direction, in order to avoid over and under-dosages especially in the overlap regions.

The study of Surucu and our phantom study were performed in the ideal situation, as the phantom is fixed and cannot move. However, real patients can have involuntary motions. In detail, the crude beam-on time required to cover the upper part with VMAT approach was found to be of around 12-15 minutes [8-13]. The time necessary for patient pre-positioning and for imaging should be added to these values (in our experience the door to door time is around 60-90 minutes) increasing the dosimetric uncertainty due to patient movements. In vivo measurements on TBI reported dose uniformity within 10% over the body [21-23]. The TMI-TMLI possibility of immobilizing the patient along the usual radiotherapy position (i.e. on the couch at skin source distance of 80-120 cm, instead of the usual 300 cm) guaranteed a better correspondence between the calculated dose on TPS and the real dose delivered to the patient as we found GAI (5%, 5 mm) > 95% in all cases. Furthermore, the GAI (5%, 3 mm) greater than 88% confirms, also, the good positioning and the reliability of the “All Body” frame for TMI-TMLI patients.

This study was focused on the upper part of TMI-TMLI (i.e. up to femurs). The lower part of the legs with the overlap region between the two plans was not considered in this study. LINACS couch moving ability, indeed, is limited to around 140 cm, requiring the patient to be positioned twice: a head-first-supine and a feet-first-supine. In our previous work [13] we evaluated the field junction robustness from consecutive fields from different isocenters from the same CT scan. The same optimization procedure could not be applied when the isocenters are derived from different CT series. Therefore this specific in-vivo dosimetry evaluation is quite particular and requires a specific analysis.

In conclusion, the delivered dose was found to be in good agreement with the planned one for the TMI-TMLI protocol. A systematic evaluation over more patients is ongoing in order to improve the data robustness.
